# Internet Access and Hypertension Management Among the Elderly Population: A Nationally Representative Cross-Sectional Survey in China

**DOI:** 10.2196/11280

**Published:** 2019-01-31

**Authors:** Yinzi Jin, Mingxia Jing, Luyu Zhang, Suhang Song, Xiaochen Ma

**Affiliations:** 1 Department of Global Health School of Public Health Peking University Beijing China; 2 Department of Public Health Shihezi University School of Medicine Xinjiang China; 3 China Center for Health Development Studies Peking University Beijing China

**Keywords:** China, health disparity, hypertension, internet

## Abstract

**Background:**

Hypertension is a rapidly growing epidemic in China. Yet, it remains inadequately controlled, especially in rural areas. The internet has shown potential for better health management in different settings; however, few studies have investigated its role in hypertension management in China.

**Objective:**

This study aims to examine the association between internet access and hypertension awareness, treatment, and control among elderly Chinese adults and to investigate whether the association between internet access and hypertension management differed between those living in urban and rural areas.

**Methods:**

We obtained data from the nationally representative survey of the China Health and Retirement Longitudinal Study in 2011. Hypertension was defined as (1) average systolic blood pressure of ≥140 mm Hg or average diastolic blood pressure of ≥90 mm Hg or (2) currently taking antihypertensive medications. The outcome assessed included hypertension awareness, treatment, and control. The key independent variable was defined as whether one had internet access at home. We performed multivariate logistic regressions for each of the 3 outcomes.

**Results:**

Among 5135 hypertensive respondents (age 62.4 [SD 9.9] years; 2351/5135, 45.78% men), 12.89% (662/5135) had internet access at home. Compared with those who had no internet access, internet access was positively associated with hypertension awareness (odds ratio [OR] 1.36, 95% CI 1.07-1.73) and treatment (OR 1.38, 95% CI 1.09-1.75), but not with control (OR 1.19, 95% CI 0.90-1.58). Internet access reduced urban-rural disparity in hypertension awareness by 9.6% (*P*=.02), treatment by 8.3% (*P*=.05), but not in control. In addition, the moderating effect of internet access on urban-rural disparities in hypertension management was larger among females. The decreased urban-rural disparities were primarily driven by that internet access improved the management level in rural areas.

**Conclusions:**

Despite the low rate of internet access among the elderly population, the internet shows its potential as a platform for achieving better hypertension management in China. Strategies for reducing the disparities in hypertension management and overall disease burden of hypertension among the elderly population might consider the internet as a platform.

## Introduction

Hypertension is a major health problem and largely contributes to morbidity and mortality worldwide [1]. In China, there is a growing epidemic of hypertension among the population as a whole and urban and rural population specifically [2]. Over the past decades, lifestyle and dietary pattern have changed substantially in rural areas owing to China’s rapid economic growth and urbanization [3]. As a result, the hypertension prevalence in the rural population is getting close to that of their urban counterparts [3]. However, most health facilities and human resources are located in urban areas [4]. Partially owing to the unequal distribution of health resources, a very pronounced gap exists in hypertension awareness, treatment, and control between urban and rural residence in China [2].

The successful management of hypertension, like optimal control of many other chronic diseases, requires early detection and sufficient compliance to medications [5]. A growing strand of literature has studied the internet as a potential platform for improved disease management. The internet provides patients opportunities for efficient information search and purchase of medication and devices, timely consulting with health providers, as well as real-time data monitoring [6-8]. Positive empirical evidence in developed countries has accumulated in the field of chronic diseases control like diabetes, respiratory disease, and cardiac disease [9-11]. However, 2 key questions remained unanswered; first, does the positive effect on chronic disease management found in other settings apply to hypertension management in China, where access to qualified health workers and effective medications is limited [12]; second, given the large urban­­-rural disparity in hypertension management in China [13], is the association between internet access and hypertension management differential between urban and rural areas? If so, is internet widening or narrowing existing urban-rural disparities of hypertension management?

To fill this gap, this study uses a nationally representative survey to examine the role of internet access in hypertension management among the elderly population in China. The objectives of this study are 2-fold as follows: (1) to examine the association between internet access and hypertension awareness, treatment and control among elderly Chinese adults and (2) to investigate whether the association between internet access and hypertension management differed between those living in urban and rural areas.

## Methods

### Data and Sample

Data for this study were obtained from the 2011 round of China Health and Retirement Longitudinal Study (CHARLS 2011). In China, CHARLS is a nationally representative longitudinal survey of adults aged ≥45 years and their spouses, including assessments of social, economic, and health circumstances of community residents [14]. CHARLS 2011 was conducted between June 2011 and March 2012 and included 17,708 participants from 450 communities in 28 provinces. All participants provided written informed consent, and survey protocols were approved by the Peking University Ethics Review Board [15].

**Figure 1:**
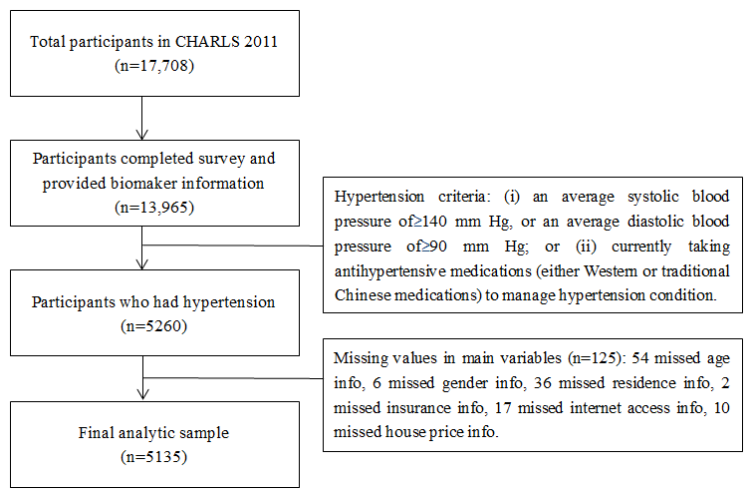
Flowchart of sample selection from the CHARLS 2011.

This study sample was selected from respondents who completed the survey and provided biomarker information of blood pressure measurement in CHARLS 2011 (n=13,965). The likelihood of nonresponse appeared to be uncorrelated with demographic, socioeconomic status characteristics [15]. Of 13,965 participants, 5260 (37.66%) were found to have hypertension (see the following subsection for detailed measurement of hypertension). Our final analytic sample included 5135 individuals who had hypertension and provided complete data for all study variables (Figure 1).

### Outcome Measures and Covariates

Blood pressure was measured 3 times at 45-second intervals using an Omron HEM-7200 monitor (Omron, Dalian Co, LTD, Dalian, China) during the daytime. Respondents were asked to relax and remain seated when measured by professionals. The final analytic blood pressure level was calculated by averaging these 3 measures. Hypertension was defined as (1) average systolic blood pressure of ≥140 mm Hg or average diastolic blood pressure of ≥90 mm Hg or (2) currently taking antihypertensive medications (either Western or traditional Chinese medications) to manage hypertension condition.

This study analyzed the following 3 hypertension management variables as outcome variables: (1) *Hypertension Awareness*, defined as an individual with hypertension who reported previous diagnosis of hypertension or simply claimed to have hypertension; (2) *Hypertension Treatment*, defined as an individual with hypertension who claimed to be taking any antihypertensive medications (either Western or traditional Chinese medications); and (3) *Hypertension Control*, defined as an individual with hypertension whose average systolic blood pressure <140 mm Hg and average diastolic blood pressure <90 mm Hg from the blood pressure measures.

Demographic and socioeconomic status covariates for this study included age (45-59, 60-69, and >70), gender (male or female), educational attainment (illiterate, part of primary school, primary school, middle school, high school or above), marital status (married, widowed, separated, divorced, or never married), and insurance status (whether or not uninsured). Family wealth was measured by the market price of the house an individual owned and currently resided. As about 16.38% (841/5135) of our sample did not own a house, their family wealth was coded as zero. Then, a categorical variable of 3 terciles was generated, which cut the sample into lower, middle, and upper tercile based on the value of their family wealth. Lifestyle covariate included smoking behavior (currently smoking or not), comorbidities covariate included the number of co-occurring chronic diseases (whether >3 co-occurring chronic diseases). The key independent variable was defined as whether one had internet access at home. Urban or rural residence was defined as the *Hukou* household registration record of the individual during the survey.

### Statistical Analysis

A descriptive analysis presented the characteristics of study participants among the full sample as a whole and by a subsample of urban and rural areas. Two multivariate logistic regression models were estimated to investigate the role of internet access in hypertension management. Model 1 regressed each outcome variable on internet access and urban residence. Model 2 added an additional regressor of the interaction term between urban residence and internet access. Both models were adjusted for covariates with a *P* ≤.05 in the descriptive analysis. Province fixed effects were added in both models to adjust for unobserved provincial-level factors. The sign of interaction term in Model 2 could be interpreted as whether internet access modified the urban-rural disparity of hypertension management [16,17].

To provide a more intuitive interpretation of the moderating effect of internet access on the urban-rural disparity of hypertension management, we computed the mean and SE of marginal effects for the interaction term in logistic regressions. We repeated our computation using full sample and subsample by gender. In addition, we calculated whether the moderating effect of internet access on urban-rural disparities was primarily driven by the fact that internet changed hypertension management levels in urban or rural areas. All analyses were conducted in Stata 14.1 (StataCorp LP).

## Results

### Characteristics of Study Participants

Data from 5135 eligible participants with hypertension aged ≥45 years (4000 rural subjects and 1135 urban subjects) were included for analysis. Table 1 provides the characteristics of these participants. Hypertension management levels were low, and among the 5135 participants, awareness, treatment, and control rate were 56.94% (2924), 49.15% (2524), and 20.29% (1042), respectively. Compared with urban participants, rural participants were younger (945/4000, 23.62%, aged ≥70 years in rural areas vs 324/1135, 28.6%, in urban; *P*<.001), comprised more females (2228/4000, 55.70% vs 556/1135, 48.99%; *P*<.001), were less educated (1497/4000, 37.42%, were illiterate in rural areas vs 144/1135, 12.69%, in urban; *P*<.001), were poorer (1160/4000, 29.0%, were in rich tercile of family wealth in rural areas vs 522/1135, 45.99%, in urban; *P*<.001), and had less access to internet at home (329/4000, 8.22% vs 333/1135, 29.34%; *P*<.001), but were less likely to be uninsured (236/4000, 5.90% vs 118/1135, 10.40%; *P*<.001). In terms of hypertension management, rural participants had lower rates of awareness (2165/4000, 54.12%, in rural vs 759/1135, 66.87%, in urban; *P*<.001), treatment (1834/4000, 45.85%, in rural vs 690/1135, 60.79%, in urban; *P*<.001), and control (734/4000, 18.35%, in rural vs 308/1135, 27.14%, in urban; *P*<.001) than their urban counterparts.

**Table 1:** Characteristics of the study participants.

Characteristics				National (n=5135), n (%)		Rural (n=4000), n (%)		Urban, (n=1135), n (%)			*P* value	
**Covariates**												
	**Age in years**									<.001		
			45-59	2153 (41.93)		1718 (42.95)		435 (38.33)				
			60-69	1713 (33.36)		1337 (33.42)		376 (33.13)				
			≥70	1269 (24.71)		945 (23.62)		324 (28.55)				
	Male sex				2351 (45.78)		1772 (44.30)		579 (51.01)			<.001
	**Education**									<.001		
			Illiterate	1641 (31.96)		1497 (37.42)		144 (12.69)				
			Part of primary school	945 (18.40)		822 (20.55)		123 (10.84)				
			Primary school	1139 (22.18)		883 (22.07)		256 (22.56)				
			Middle school	913 (17.78)		612 (15.30)		301 (26.52)				
			High school or above	497 (9.68)		186 (4.65)		311 (27.40)				
	**Marital status**									.32		
			Married	4220 (82.18)		3269 (81.72)		951 (83.79)				
			Widowed	801 (15.60)		647 (16.18)		154 (13.57)				
			Separated, divorced, or never married	114 (2.22)		84 (2.10)		30 (2.64)				
	**Market price of house**									<.001		
			Poor tercile	1803 (35.11)		1415 (35.38)		388 (34.19)				
			Middle tercile	1650 (32.13)		1425 (35.62)		225 (19.82)				
			Rich tercile	1682 (32.76)		1160 (29.00)		522 (45.99)				
	Uninsured				354 (6.89)		236 (5.90)		118 (10.40)			<.001
	>3 co-occurring chronic diseases				1180 (36.61)		1415 (35.38)		465 (40.97)			.001
	Currently smoke				2007 (39.08)		1556 (38.90)		451 (39.74)			.61
Internet access at home as key independent variable				662 (12.89)		329 (8.22)		333 (29.34)			<.001	
**Hypertension management**												
		Awareness		2924 (56.94)		2165 (54.12)		759 (66.87)			<.001	
		Treatment		2524 (49.15)		1834 (45.85)		690 (60.79)			<.001	
		Control		1042 (20.29)		734 (18.35)		308 (27.14)			<.001	

### Association Between Internet Access and Hypertension Management

**Figure 2:**
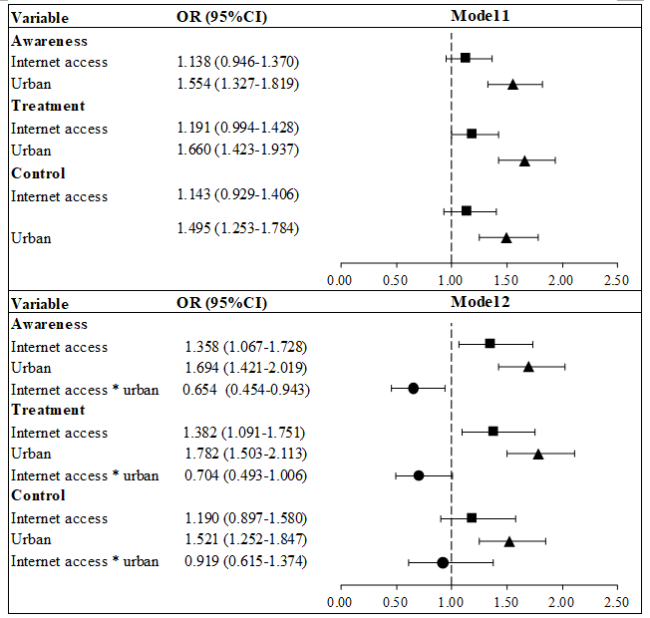
Multivariate logistic regressions on multivariate relationship of internet access and urban residence to hypertension management.

Figure 2 plots the odds ratios (ORs) of multivariate logistic regressions. Without adding the interaction term between urban residence and interaction access (Model 1), internet access at home was positively associated with all of the 3 hypertension management outcomes; however, none of the coefficients was statistically significant: hypertension awareness (OR 1.14, 95% CI 0.95-1.37; *P*=.17), treatment (OR 1.19, 95% CI 0.99-1.43; *P*=.06), and control (OR 1.14, 95% CI 0.93-1.41; *P*=.21). In addition, we found significant and notable urban-rural disparities in hypertension awareness (OR 1.55, 95% CI 1.33-1.82; *P*<.001), treatment (OR 1.66, 95% CI 1.43-1.94; *P*<.001), and control (OR 1.50, 95% CI 1.25-1.78; *P*<.001).

After adding the interaction term (Model 2), the positive associations between internet access and hypertension management became statistically significant and with larger coefficient sizes in hypertension awareness (OR 1.36, 95% CI 1.07-1.73; *P*=.01) and treatment (OR 1.38, 95% CI 1.09-1.75; *P*=.007), but not in hypertension control (OR 1.19, 95% CI 0.90-1.58; *P*=.23). In terms of urban-rural disparities, the coefficient sizes were even larger in all 3 outcomes: hypertension awareness (OR 1.69, 95% CI 1.42-2.02; *P*<.001), treatment (OR 1.78, 95% CI 1.50-2.11; *P*<.001), and control (OR 1.52, 95% CI 1.25-1.85; *P*<.001).

### Association Between Internet Access and Urban-Rural Disparity of Hypertension Management

The ORs of the interaction term were <1 for each outcome, indicating that internet access reduced the urban-rural gap in hypertension management, although the OR of the interaction term in hypertension control was statistically insignificant. Specifically, the OR of the interaction term between internet access and urban residence in hypertension awareness was 0.65 (95% CI 0.45-0.94; *P*=.02), indicating that the effect of internet access on being aware of hypertensive status for urban residents was 35% smaller than that on rural residents. Similar patterns were also observed for hypertension treatment (OR 0.70, 95% CI 0.49-1.01; *P*=.05) and control (OR 0.92, 95% CI 0.62-1.37; *P*=.68).

Tables 2, 3, and 4 provide a more intuitive interpretation of whether internet access modified the urban-rural disparities on hypertension management by presenting the mean marginal effects of the interaction term between urban residence and internet access. Internet access reduced the urban-rural disparities on hypertension awareness by 9.6% (*P*=.02), hypertension treatment by 8.3% (*P*=.05). We observed no statistically significant change in hypertension control. In addition, these interaction effects were disproportionally and more significantly benefiting female participants; internet access reduced the urban-rural disparities in hypertension awareness by 14.7% (*P*=.01), in hypertension treatment by 14.5% (*P*=.01) among female participants.

The decreased urban-rural disparities in hypertension awareness and treatment associated with internet access were primarily driven by the significant improvement of management level in rural areas among those with internet access at home compared with those without internet access at home. However, the management level remained constant in urban areas between those with and without internet access at home (Tables 5, 6, and 7).

**Table 2:** Marginal effects of the interaction term between urban residence and internet access for the awareness hypertension.

Population	Coefficient	SE	*P* value
Total (N=5135)	−0.096^a^	0.042	.02
Male (N=2351)	−0.046^a^	0.061	.45
Female (N=2784)	−0.147^a^	0.057	.01

^a^All values adjusted for 6 covariates with a *P* ≤.05 in Table 1: age, gender, educational level, uninsured, the market price of the house, >3 co-occurring chronic diseases. Province fixed effects were also adjusted.

**Table 3:** Marginal effects of the interaction term between urban residence and internet access for the treatment of hypertension.

Population	Coefficient	SE	*P* value
Total (N=5135)	−0.083^a^	0.042	.05
Male (N=2351)	−0.012^a^	0.062	.83
Female (N=2784)	−0.145^a^	0.058	.01

^a^All values adjusted for 6 covariates with a *P* ≤.05 in Table 1: age, gender, educational level, uninsured, the market price of the house, and >3 co-occurring chronic diseases. Province fixed effects were also adjusted.

**Table 4:** Marginal effects of the interaction term between urban residence and internet access for the control of hypertension.

Population	Coefficient	SE	*P* value
Total (N=5135)	−0.010^a^	0.036	.79
Male (N=2351)	0.075^a^	0.052	.15
Female (N=2784)	−0.091^a^	0.050	.07

^a^All values adjusted for 6 covariates with a *P* ≤.05 in Table 1: age, gender, educational level, uninsured, the market price of the house, and >3 co-occurring chronic diseases. Province fixed effects were also adjusted.

**Table 5:** Estimated hypertension awareness by urban residence and internet access.

Variable	Rural^a^	Urban^a^
No Internet	0.43 (0.03)	0.54 (0.02)
Internet	0.49 (0.03)	0.49 (0.03)
*P* value	.03	.10

^a^All values adjusted for 6 covariates with a *P* ≤.05 in Table 1: age, gender, educational level, uninsured, the market price of the house, and >3 co-occurring chronic diseases. Province fixed effects were also adjusted.

**Table 6:** Estimated hypertension treatment by urban residence and internet access.

Variable	Rural^a^	Urban^a^
No Internet	0.34 (0.03)	0.47 (0.02)
Internet	0.41 (0.03)	0.44 (0.03)
*P* value	.02	.27

^a^All values adjusted for 6 covariates with a *P* ≤.05 in Table 1: age, gender, educational level, uninsured, the market price of the house, >3 co-occurring chronic diseases. Province fixed effects were also adjusted.

**Table 7:** Estimated hypertension control by urban residence and internet access.

Variable	Rural^a^	Urban^a^
No Internet	0.15 (0.03)	0.22 (0.02)
Internet	0.17 (0.02)	0.22 (0.03)
*P* value	.44	.89

^a^All values adjusted for 6 covariates with a *P* ≤.05 in Table 1: age, gender, educational level, uninsured, the market price of the house, >3 co-occurring chronic diseases. Province fixed effects were also adjusted.

## Discussion

### Principal Findings

To the best of our knowledge, this study was the first to examine the role of internet access in hypertension management among the elderly population in China. Hypertension is a leading cause of mortality and disability around the world [18]. In China, it accounted for >2.5 million deaths (almost one-third of total deaths) and 15% of total disability-adjusted life-years in 2013, mainly from stroke and ischemic heart disease [18,19]. Over the past decade, the proportion of population connected to the internet has been growing exponentially [20], and researchers in the field of health promotion have been quick to capitalize on the internet to promote changes in health behavior in many settings [21]. Taking advantage of the population-based nationally representative survey CHARLS 2011, we were able to investigate whether the positive effect of the internet on disease management found in other settings still holds in the context of China, where fast economic growth and population aging are happening simultaneously, and more importantly, whether the internet has a moderating effect on the existing urban-rural gap in hypertension management.

In this study, several key findings were highlighted. First, internet access at home was associated with better hypertension management among elderly Chinese adults. Previous studies have found a positive effect of the internet on disease management in different settings [22-26]. For example, one randomized trial proved that internet-based chronic disease self-management program was effective in changing health-related behaviors and improving the health status of patients with chronic diseases [7]. However, few have investigated whether this positive effect still holds in developing context such as China. The positive association between internet access and hypertension management in this study was of practical importance in the sense that China is facing a huge public health crisis of hypertension in which sufficient awareness and compliance to medications are the necessary steps to achieve adequate control.

Second, the finding that internet access was associated with decreased urban-rural disparities in hypertension management was encouraging. The existing gap in hypertension awareness, treatment, and control between urban and rural areas found in this study paralleled that of a recent nationwide population-based epidemiology study of hypertension prevalence and management among Chinese adults [2]. In this study, we were able to use a rich individual-level dataset and examine the association between internet access and urban-rural disparities in hypertension management adjusted for demographic characteristics, socioeconomic status, comorbidity, and lifestyle factors. The magnitudes of 9.6% decrease in hypertension awareness and 8.3% decrease in hypertension treatment were considered to be substantial compared with other studies measuring urban-rural disparities in hypertension management among Chinese patients [13,27]. A well-documented contributor to China’s urban-rural disparities in health, including hypertension management, is the unequal distribution of health workforce [28]. Efforts to cope with the shortage of health care workers in rural areas have been slow, especially at primary care level [29]. Our finding provided suggestive evidence that the internet could help to cope with the limited access to quality care in rural areas. More broadly, our results might shed lights to improve health equity in China [30].

Third, a significant and substantial reduction in urban-rural disparity of hypertension awareness and treatment did not translate into the reduction of hypertension control; therefore, improving both is necessary but not sufficient to achieve the optimal control of hypertension [2]. Hypertension control often requires multiple medications, yet in rural China, the availability and affordability to antihypertensive drugs were limited [31]. A recent national study showed that less than one-third of primary health care institutions stocked guideline-recommended and low-cost antihypertensive medications, and unfortunately the deficiencies were even worse in rural areas [31].

Exploring clear mechanisms in explaining the association between internet access and decreased urban-rural disparities in hypertension management was beyond the scope of this study. Nevertheless, we found the association was more notable and statistically significant among female participants. This was in line with a series of studies emphasizing the fact that female patients are more responsive to hypertension management interventions [32,33]. Future studies should focus on understanding channels through which internet access works on improving hypertension management. In addition, decreased urban-rural disparities in hypertension management were primarily driven by the fact that internet access improved management levels in rural areas. This is an encouraging finding, highlighting the importance of improving the hypertension management in rural areas to reduce the burden of hypertension [34]. Furthermore, the results of this study were consistent with previous studies that showed better health management by the internet-based health care models in rural areas and less-developed regions [35,36].

### Limitations

This study has several limitations. First, the observational nature of our study limited our ability to draw any causal inference from our findings. The results should not be interpreted as the effect of internet access on reducing urban-rural disparities in hypertension management. Rather, the association found in this study underscored the need for research to capitalize on new technologies to mitigate the disease burden of hypertension. Second, measuring blood pressure 3 times in the CHARLS study might not lead to the most accurate hypertension diagnosis. The medical literature has confirmed that the 24-hour ambulatory blood pressure monitoring to be the best assessment of hypertension [37]. However, given the large-scale, population-based survey design of CHARLS, the 24-hour ambulatory blood pressure monitoring might not be cost-effective or feasible in implementation. Third, in the 2011 round of CHARLS survey, only 29% and 8% household had internet access in urban and rural areas. This low rate of internet access among our sample population might limit us to generate policy relevance. Although we did not have data of latest internet access among the elderly population, it was reported that internet availability has improved markedly across the country over the past few years [20]. Nonetheless, this lower internet access rate might make our estimates of the association between internet access and reduced urban-rural disparities in hypertension management conservative. Fourth, although the 2.4% nonresponse rate could be considered as low given the large-scale survey of CHARLS, our nonresponse analysis was only limited to comparing hypertension outcome variables between response and nonresponse groups. We have to acknowledge that the nonresponse analysis provided by CHARLS was based on the whole sample, which might not apply to our sample of hypertensive participants. However, because those nonresponses in our study did not complete the survey and only provided biomarker information, we were only to compare the hypertension outcome variables and found nonresponse was not associated with hypertension awareness, treatment, and control (see Multimedia Appendix 1). Last but not least, even if we used a nationally representative survey of the elderly population in China, the generalizability of our results should be limited to the context of China. Given the rapid pace of population aging in China, we believed that our results among elderly adults were of policy importance to that specific population in China.

### Conclusions

Hypertension is becoming a public crisis in China and around the globe. Using a nationally representative survey of Chinese elderly, we found that internet access at home was associated with better hypertension management, and internet access reduced the urban-rural gap in hypertension management outcomes. Despite the low rate of internet access among the elderly population, the internet shows its potential as a platform for achieving better hypertension management in China. Longitudinal studies on the internet and hypertension management, because internet facilities have improved significantly in recent years, are needed. Given the growing epidemic of hypertension and the rapid pace of population aging in China, we believe that this study sheds lights on designing polices for achieving optimal hypertension management and health equity in China. Strategies for reducing the disparities in hypertension management and overall disease burden of hypertension might consider the internet as a platform for disease management.

## Appendix

Multimedia Appendix 1 Comparison of hypertension management outcomes between response group and non-response group.

## Abbreviations

CHARLS: China Health and Retirement Longitudinal Study
OR: odds ratio
