# Supplementary material for: Internet Access and Hypertension Management Among the Elderly Population: A Nationally Representative Cross-Sectional Survey in China
Source: J Med Internet Res. 2019 Jan 31;21(1):e11280. doi: 10.2196/11280 (PMC6374727; doi:10.2196/11280)
Supplement: Multimedia Appendix 1 [file jmir_v21i1e11280_app1.pdf]

Multimedia Appendix 1 Comparison of hypertension management outcomes between response group and non-response group

	Response (n = 5135)	Non-response (n = 125)	P-value [a]
Proportion of Awareness (SD)	57.21 (6.92)	57.72 (4.47)	0.909
Proportion of Treatment (SD)	49.15 (6.98)	44.00 (4.45)	0.255
Proportion of Control (SD)	20.29 (5.61)	24.00 (3.83)	0.309

[a] T-test was performed between the response group and non-response group.
